# Human Serum Mediated Bacteriophage Life Cycle Switch in *Aggregatibacter actinomycetemcomitans* Is Linked to Pyruvate Dehydrogenase Complex

**DOI:** 10.3390/life13020436

**Published:** 2023-02-03

**Authors:** Gaoyan Grace Tang-Siegel

**Affiliations:** Department of Molecular Physiology and Biophysics, College of Medicine, University of Vermont and State Agricultural College, Burlington, VT 05405, USA; gaoyan.tang-siegel@uvm.edu; Tel.: +1-(802)-656-2519

**Keywords:** bacteriophage, lysogen, life cycle, Gram-negative bacterium, capnophile, facultative anaerobe, periodontal diseases, HACEK microorganisms, human serum, pyruvate dehydrogenase complex (PDHc)

## Abstract

Antimicrobial resistance is rising as a major global public health threat and antibiotic resistance genes are widely spread among species, including human oral pathogens, e.g., *Aggregatibacter actinomycetemcomitans*. This Gram-negative, capnophilic, facultative anaerobe is well recognized as a causative agent leading to periodontal diseases, as well as seriously systemic infections including endocarditis. *A. actinomycetemcomitans* has also evolved mechanisms against complement-mediated phagocytosis and resiliently survives in serum-rich in vivo environments, i.e., inflamed periodontal pockets and blood circulations. This bacterium, however, demonstrated increasing sensitivity to human serum, when being infected by a pseudolysogenic bacteriophage S1249, which switched to the lytic state as a response to human serum. Concomitantly, the pyruvate dehydrogenase complex (PDHc), which is composed of multiple copies of three enzymes (E1, E2, and E3) and oxidatively decarboxylates pyruvate to acetyl-CoA available for tricarboxylic acid (TCA) cycle, was found up-regulated 10-fold in the bacterial lysogen after human serum exposure. The data clearly indicated that certain human serum components induced phage virion replication and egress, resulting in bacterial lysis. Phage manipulation of bacterial ATP production through regulation of PDHc, a gatekeeper linking glycolysis to TCA cycle through aerobic respiration, suggests that a more efficient energy production and delivery system is required for phage progeny replication and release in this in vivo environment. Insights into bacteriophage regulation of bacterial fitness in a mimic in vivo condition will provide alternative strategies to control bacterial infection, in addition to antibiotics.

## 1. Introduction

Antimicrobial resistance is rising as a major global public health threat [1], and antibiotic resistance genes are widely spread, including human oral microbiome [2]. Antibiotic resistant strains have been identified from the Gram-negative bacterium *Aggregatibacter actinomycetemcomitans* [3,4], with recognized antibiotic resistance mechanisms including mobile rRNA methylase genes [5] and the multidrug efflux pump system AcrAB-TolC [6,7]. This microorganism is found in approximately 20% of the population [8], and causes the aggressive form of periodontitis [9]. Periodontitis is characterized by inflammatory periodontia, and inflammation increases the permeability of blood vessels and allows bacteria to exude from the periodontal pocket into the blood circulation to initiate systemic infections, including infective endocarditis and neuron inflammatory comorbidities [10,11]. *A. actinomycetemcomitans* is well recognized for expressing multiple virulence factors that exacerbate the progression of both oral and systemic infections, including bundle fimbriae [12,13], non-fimbrial adhesins of EmaA [14,15,16,17,18] and Aae [19,20], two important exotoxins: leukotoxin [21,22] and cytolethal extending toxin [23], and at least seven different lipopolysaccharide endotoxins [24,25,26,27,28]. Importantly, this pathobiont is resilient in vivo due to its resistance to killing by serum complement mediated phagocytosis [29,30,31,32].

We, however, have identified a pseudolysogenic phage S1249, which increases the sensitivity of *A. actinomycetemcomitans* to human serum [32,33,34,35]. Over 20% of phage genes were up-regulated at least 10-fold with a concomitant increase in the kinetics of phage egress, after the exposure of its lysogen to human serum [33,35], including a cluster of phage regulatory factors [35]. Interestingly, sera from other animals, including equines, do not stimulate the same response [32,33,35]. Bacteriophages have been emerging as alternative therapeutics to treat certain bacterial infection to combat the rising antibiotic resistance [36], as well as a potential delivery platform for future vaccine developments [37]. Therefore, understanding phage infection and replication behaviors in a mimicked in vivo environment, including serum rich blood circulation, is necessary.

In this study, we used a newly generated lysogenic strain of phage S1249 as a tool to investigate how in vivo growth environment may affect the life cycle determination of the phage. We focused on bacterial host membrane-associated protein changes during the phage life cycle switch from the lysogenic to the lytic state after exposure to human serum. Blue native polyacrylamide gel electrophoresis (BN-PAGE) and the liquid chromatography/mass spectrometry (LC/MS) analyses clearly demonstrated an over 10-fold increase of detected protein of the pyruvate dehydrogenase complex (PDHc), which is composed of pyruvate dehydrogenase (E1), dihydrolipoamide acetyltransferase (E2), and dihydrolipoyl dehydrogenase (E3), in the lysogen grown in the presence of human serum. Importantly, the detected PDHc level peaked approximately the same time when virions released. PDHc is the central gatekeeper in glucose metabolism in both mammals and Gram-negative bacteria [38], which links the glycolytic pathway to the tricarboxylic acid (TCA) cycle to produce ATP through aerobic respiration by converting pyruvate into acetyl-coenzyme A (acetyl-CoA) [39,40].

*A. actinomycetemcomitans* is a capnophilic, facultative anaerobe and requires 5% to 10% carbon dioxide for its growth. This periodontal pathogen can produce energy through either aerobic respiration at the presence of oxygen, e.g., occasional presence on the surface of oral mucosa; or anaerobic fermentation using fumarate as an electron acceptor, e.g., in the periodontal pocket, which allows itself to adapt to different in vivo habitats [39,41]. The significant up-regulation of PDHc in the lysogenic bacterial strain, but not the phage-naïve strain, when cells were exposed to human serum, suggests that an increased production and delivery of ATP, through aerobic respiration, is required for bacteriophage virion replication and egress in the in vivo serum-rich environment.

## 2. Materials and Methods

### 2.1. Phage and Bacterial Strain Preparation

All bacterial and bacteriophage strains were stored in a −80 °C freezer. *A. actinomycetemcomitans* was routinely recovered from the freezer using TSBYE agar, containing 3% trypticase soy broth, 0.6% yeast extract, and 1.5% agar (Becton Dickinson and Company, Franklin Lakes, NJ, USA) and incubated statically in a 37 °C incubator with 5% humidified carbon dioxide atmosphere for each experiment. The phage-naïve *A. actinomycetemcomitans* strain IDH84 of serotype c [14,35] and its derived lysogenized strain IDH84/S1249 were used for this investigation. The strain IDH84/S1249 was generated by infecting strain IDH84 with the isolated bacteriophage S1249, which demonstrated phage DNA integration into the bacterial chromosome [35].

*Aggregatibacter* phage S1249 was isolated from its originally identified clinical lysogenic strain D11S-1 [32,35,42], using a modified protocol described by Tang-Siegel et al. [35]. Briefly, the overnight culture of 10 mL bacterial lysogen grown at 37 °C with 5% humidified CO_2_ atmosphere using TSBYE was diluted in 100 mL, grown for two doubling-time (6 h), and exposed to 0.5 μg/mL mitomycin C from *Streptomyces caespitosus* (M0503; Sigma-Aldrich, St Louis, MO, USA) for 30 min. Bacterial cells were then pelleted at 8000× *g*, 4 °C for 10 min, washed twice with fresh TSBYE broth, resuspended in the same volume of TSBYE broth. After 12 h recovering at 37 °C with 5% humidified CO_2_ atmosphere for virion replication and egress, the culture suspension was centrifuged at 16,000× *g*, 4 °C for 30 min, and the supernatant was collected, filtered with 0.22 μm to remove any contaminated bacteria, and ultra-centrifuged at 100,000× *g*, 4 °C for 1 h to collect phages. The isolated phage was resuspended and stored in phage suspension medium (100 mM NaCl, 8mM MgSO4.7H_2_O, 0.01% gelatin, 50 mM Tris-HCL), confirmed without bacterial contamination by culture, and aliquots were kept at −80 °C before use [35].

### 2.2. Growth Comparison after Mitomycin C Exposure

The phage-naïve strain IDH84 and the lysogenized strain IDH84/S1249 were recovered from −80 °C using TSBYE agar plates. A single colony of each strain was inoculated into 10 mL TSBYE broth and grown overnight. The overnight culture of each strain will be diluted using fresh TSBYE and adjusted to the same optical density (OD_495_ = 0.2), which was equivalent to 5 × 10^8^ cells/mL, grown for one doubling-time (3 h). Each strain was prepared in duplicates: one was exposed to 0.5 μg/mL mitomycin C for 30 min (IDH84_Mit, IDH84/S1249_Mit), and the second sample was used as a negative control without exposure to mitomycin C (IDH84_c, IDH84/S1249_c). After induction, cells were pelleted using 8000× *g* at 4 °C for 10 min, washed twice with fresh TSBYE broth, resuspended in the same volume of TSBYE broth, incubated at 37 °C with 5% humidified CO_2_ atmosphere. The recovery of bacteria was monitored by measuring OD_495_ values. Data were collected from triplicate experiments, analyzed using ANOVA through GraphPad Prism 9 and, *p* < 0.05 was considered statistically significant.

### 2.3. Transmission Electron Microscopy (TEM) Analysis

Phage samples were prepared using the generated lysogenic strain IDH84/S1249 [35]. The lysogen was grown to the mid logarithmic phase, exposed to mitomycin C for phage induction. Bacterial cells were then pelleted at 8000× *g* for 10 min, washed twice with fresh TSBYE, resuspended in the same volume of broth. After overnight recovery, the culture suspension was directly centrifuged at 16,000× *g* for 30 min, and the supernatant was collected, ultra-centrifuged for 100,000× *g* to concentrate released phages for TEM using a modified negative stain protocol [35]. The phage sample was resuspended in phage suspension buffer, diluted to proper concentrations. Approximately, 5 μL of phage sample was loaded onto a 200-mesh carbon-coated nickel grid, washed using 6 μL filter-sterile, de-ionized water, negatively stained with 5 μL of 2% uranyl acetate for 1 min, and the remained stain liquid was absorbed at the edge of the grid using a filter paper. Grids were air dried overnight and imaged using a JOEL electron microscope (Peabody, MA, USA).

### 2.4. Bacteriophage Plaque Assays

The bacteriophage plaque assay was performed using three different preparations of the bottom hard agar layers for providing nutrition: TSBYE, TSBYE mixed with 50% heat-inactivated equine serum (262-500; Quad Five, Ryegate, MT, USA) in volume, and TSBYE mixed with 50% heat-inactivated, pooled, human male, type AB serum (H3667; Sigma-Aldrich, St Louis, MO, USA). The bottom layers were prepared with a final concentration of 1.5% agar, and approximately 6 mL of medium was poured into a petri dish plate (100 mm × 15 mm), kept warm in a 37 °C incubator with 5% humidified CO_2_ before pouring the top bacterium and phage soft agar mixture.

A phage naïve serotype c strain of A. actinomycetemcomitans IDH84 [35] was chosen for the plaque assay. The bacterium was recovered from −80 °C using TSBYE agar plates, and a single colony was inoculated into 10 mL TSBYE broth, grown overnight and reached the stationary phase with approximately 2.0 × 10^9^ colony forming units (CFUs)/mL. Stationary phase bacterial cells were diluted in 1:10, grown one doubling time (3 h). A total of 300 μL of bacteria in the early logarithmic phase with a concentration of 4.0 × 10^8^ CFUs/mL in TSBYE was used for each plaque assay. The isolated phage S1249 was diluted in phage suspension medium [35] with a concentration of approximately 2.0 × 10^3^ plaque forming units (PFUs)/mL. A total of 300 μL bacterial suspension (~1.2 × 10^8^ CFUs) in TSBYE and 100 μL phage suspension (~2.0 × 10^2^ PFUs) were mixed first and preincubated at room temperature for 30 min for phage absorption. For preparing the top soft agar for the phage plaque assay, 3 mL of 45 °C prewarmed TSBYE with 0.7% agar and preincubated 400 μL mixture of bacteria and phages were combined by gentle and quick tilting the tube for a few times, before spreading evenly onto the bottom agar [43,44]. Plates were inverted and incubated at 37 °C with 5% humidified CO_2_ for 24–48 h, after the top soft agar completely solidified. Plaques formed on three different bottom agars were analyzed using ANOVA through GraphPad Prism Version 9.5.0 (525), Boston, MA, USA and, *p* < 0.05 was considered statistically significant.

### 2.5. Blue Native-Polyacrylamide Gel Electrophoresis (BN-PAGE)

Bacteria were recovered from −80 °C using TSBYE agar plates, and a single colony was inoculated into 10 mL fresh TSBYE broth, grown overnight, and bacteria were inoculated from TSBYE agar to liquid TSBYE culture medium using polystyrene culture tubes without serum and grown overnight, and the cells were subsequently exposed to fresh TSBYE media with and without 50% human serum. Cells were collected every 3 h for protein biochemical analysis. The collected bacterial samples were washed twice with sterile phosphate-buffered saline (PBS: 10 mM sodium phosphate, 150 mM sodium chloride) at pH 7.4, and re-suspended in proper volume of PBS. Approximately equal numbers of bacterial cells from each sampling were treated with a mild, non-ionic detergent, n-dodecyl-β-D-maltoside (DDM, Invitrogen, Carlsbad, CA, USA) to solubilize bacterial membrane proteins in their native forms [45,46]. Cells were incubated with 1% DDM on ice for 15 min, followed by centrifugation at 16,000× *g*, 4 °C for 40 min. The collected supernatant samples containing DDM-solubilized membrane protein complexes were loaded into a 3–12% Bis-Tris gradient gel and separated by running the BN-PAGE (ThermoFisher Scientific, Waltham, MA, USA) at 150 V, 4 °C for 1 h, followed by 250 V for 90 min. Proteins separated by BN-PAGE were fixed with 50% methanol, 10% acetic acid for 10 min, further stained with colloidal blue stain (Invitrogen, Carlsbad, CA, USA) with agitation for 3 h, and de-stained in deionized water for 16 h. Bands of interest were excised for liquid chromatography/mass spectrometry (LC/MS) analysis. All BN-PAGE analyses were based on samples collected from duplicate experiments.

### 2.6. Liquid Chromatography/Mass Spectrometry (LC/MS) Analysis

Identified protein bands showing differences in the lysogenic strain, particularly in the growth condition of human serum, based on the BN-PAGE analysis were excised for the LC/MS analysis, following an in-gel trypsin digestion protocol. Briefly, each excised protein band was cut into 1 mm cubes, destained using 25 mM NH_4_HCO_3_ and acetonitrile, treated with 10 mM dithiothreitol (DTT) in 100 mM NH_4_HCO_3_ for reducing disulfide bonds, alkylated using 55 mM iodoacetamide in 100 mM NH_4_HCO_3_, and digested with trypsin. Final yielded peptides were extracted using 5% formic acid. Samples were analyzed using Thermo Q Exactive Plus mass spectrometer (ThermoFisher Scientific, Waltham, MA, USA). Obtained MS spectra were searched against the databases of *A. actinomycetemcomitans* strain D11S-1 (https://www.ncbi.nlm.nih.gov/nuccore/CP001733.2 accessed on 27 June 2016) and *Aggregatibacter* phage S1249 (https://www.ncbi.nlm.nih.gov/nuccore/GQ866233.1 accessed on 31 January 2014), as well as Homo sapiens (https://www.uniprot.org/proteomes/UP000005640 accessed in 1 July 2014) using Proteome Discoverer 2.5 (Thermo Electron, San Jose, CA, USA). The measurement accuracy used ±10 ppm peptide for internal calibration and 0.02 Da for MS/MS tolerance. Carboxymethylation of cysteines was set as a fixed modification and oxidation of methionine was set as a dynamic modification. Up to two missed tryptic peptide cleavages were considered, and three maximum dynamic modifications were allowed per peptide. The fixed value peptide spectrum match (PSM) validator node is included in the workflow to limit the false positive rate lower than 1%.

The obtained data from Plasma desorption (PD) were consolidated using Scaffold (version Scaffold 5.1.1, Proteome Software Inc., Portland, OR, USA). Identified peptides were only considered if they reached >95.0% probability with at least one identified peptide, based on the peptide prophet algorithm with Scaffold delta-mass correction [47,48]. Proteins that contained similar peptides and could not be differentiated based on LC/MS analysis alone were grouped to satisfy the principles of parsimony. Protein peptides of interest were semi-quantitatively analyzed based on total PSMs collected from two independent experiments [22]. Only peptides with a minimum length of six amino acids were considered for identification. Identifications of peptides were also validated by manual inspection of the mass spectra. The LC/MS analyses were duplicated in the proteomics core facility located at the University of Vermont.

## 3. Results

Our earlier work has demonstrated that phage S1249 actively switches between truly lysogenic and pseudolysogenic states in its bacterial hosts [35]. In the lysogenic state, phage DNA integrates into bacterial chromosome between the genes encoding for cold-shock DNA-binding domain-containing protein (*csp*) and glutamyl-tRNA synthetase (*gltX*). In the pseudolysogenic state, phage DNA does not integrate into bacterial chromosome and forms a circular DNA structure in the cells [35,42]. These two genotypes have been demonstrated in both the generated lysogenic strain IDH84/S1249, as well as the originally identified lysogenic strain D11S-1 [35].

### 3.1. Differential Responses of the Lysogen and Its Isogenic, Phage-Naïve Strain to Mitomycin C

**Figure 1:**
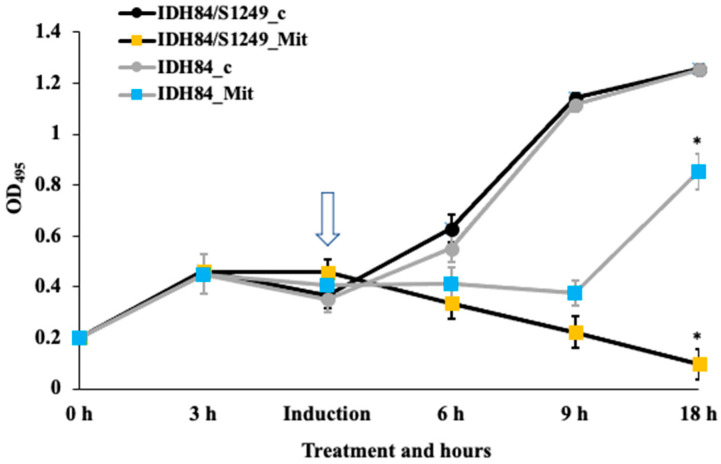
Different responses of phage-naïve vs. lysogenic strains to mitomycin C. The phage-naïve strain IDH84 demonstrated significant recovery 12 h after mitomycin C treatment (shown by the arrow). The lysogen IDH84/S1249, however, did not show any sign of recovery. IDH84/S1249_c and IDH84_c: negative control without exposure to mitomycin C; IDH84/S1249_Mit and IDH84_Mit: phage induction by exposure to mitomycin C. (* *p <* 0.05).

The current study demonstrated that phage S1249 in the newly infected strain IDH84/S1249 was also inducible using mitomycin C. After induction, the phage infected IDH84/S1249 did not show any sign of recovery within a 24-h period, due to the dominant egress of induced S1249 after exposure to mitomycin C (*p <* 0.05) (Figure 1). In contrast, the isogenic, phage-naïve strain IDH84 significantly demonstrated recovery, approximately 12 h after mitomycin C exposure (Figure 1).

### 3.2. Aggregatibacter Phage S1249 in the Lytic State Demonstrated under TEM

**Figure 2:**
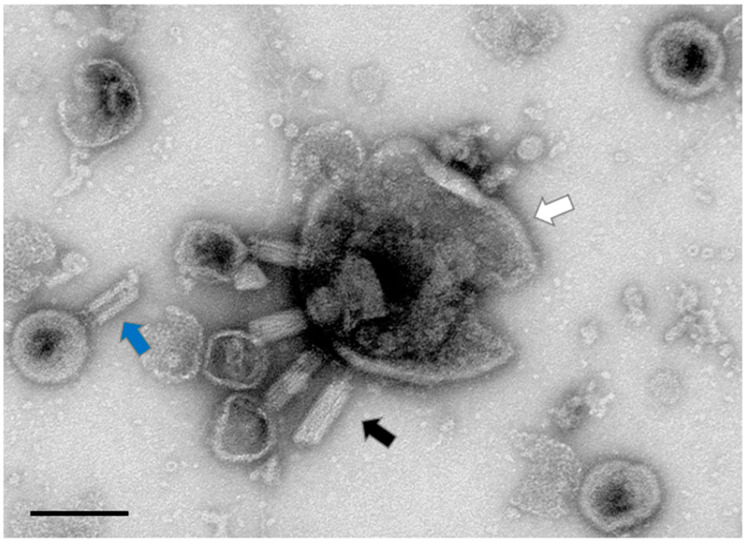
*Aggregatibacter* phage S1249 in the lytic state demonstrated by uranyl acetate negatively stained TEM. Pseudolysogenic phage S1249 entered the lytic state in the strain IDH84/S1249 (pointed by the black arrow) and lysed the bacterium for egress. A portion of the *A. actinomycetemcomitans* cell that was burst with at least four phages (pointed by the white arrow), and the central rigid tube structure of the phage tail (pointed by the blue arrow) are also illustrated. Scale bar: 100 nm.

Further examination of the lysogen IDH84/S1249 after mitomycin C induction using uranyl acetate negatively stained TEM demonstrated that the phage entered lytic cycle (Figure 2). Fifteen hours after mitomycin exposure, bacterial cells showed no indication of recovery, with continuous decline of OD (Figure 1) indicating a continuation of dominant bacterial lysis.

### 3.3. Plaque Assays Demonstrated Lytic Phage S1249 in the Presence of Human Serum

**Figure 3:**
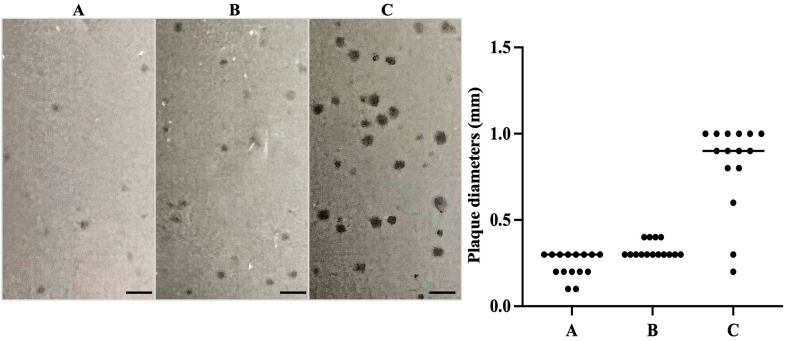
Phage plaque assay demonstrated that clear plaques formed after exposure to human serum, indicating a lytic life cycle. Approximately 1.2 × 10^8^ CFUs of *A. actinomycetemcomitans* mixed with 2.0 × 10^2^ PFUs of phage S1249 were grown in the top soft agar layer. The bottom agar layers were prepared using either (**A**) TSBYE, (**B**) TSBYE with 50% equine serum, or (**C**) TSBYE with 50% human serum. Clear, 3- to 4-fold larger plaques (0.8–1.0 mm) were formed in the presence of human serum indicating a lytic state, compared to smaller, turbid plaques (0.2–0.3 mm) indicating a lysogenic state on the agar of TSBYE or TSBYE with equine serum (*p* < 0.01). Scale bar: 2 mm.

Our early study demonstrated that the growth of generated lysogen IDH84/S1249 was significantly inhibited in the presence of human serum, showing 63% decrease in cell viability after 22 h exposure to human serum [35]. In addition, most active transcribed phage genes, when exposed to human serum, include two regions that mainly encode phage structure proteins and regulatory factors [35]. Further characterization of this phage responding to human serum in the plaque assay using the phage to infect phage-naïve strain IDH84 and exposure to three nutrients as bottom layers: TSBYE, TSBYE with 50% equine serum, or TSBYE with 50% human serum (Figure 3) demonstrated a transition from turbid plaques with diameters of ~0.2–0.4 mm formed on either TSBYE (Figure 3A) or equine serum agar (Figure 3B), indicating a lysogenic state to clear, larger plaques of approximately 0.8–1.0 mm formed on human serum agar (Figure 3C, *p* < 0.01). The phage plaque assay data lent additional support to our thesis that the life cycle switch of this phage occurs in the presence of certain human serum components.

### 3.4. Membrane Associated Proteins of Aggregatibacter Phage S1249 Lysogen Grown in Human Serum

**Figure 4:**
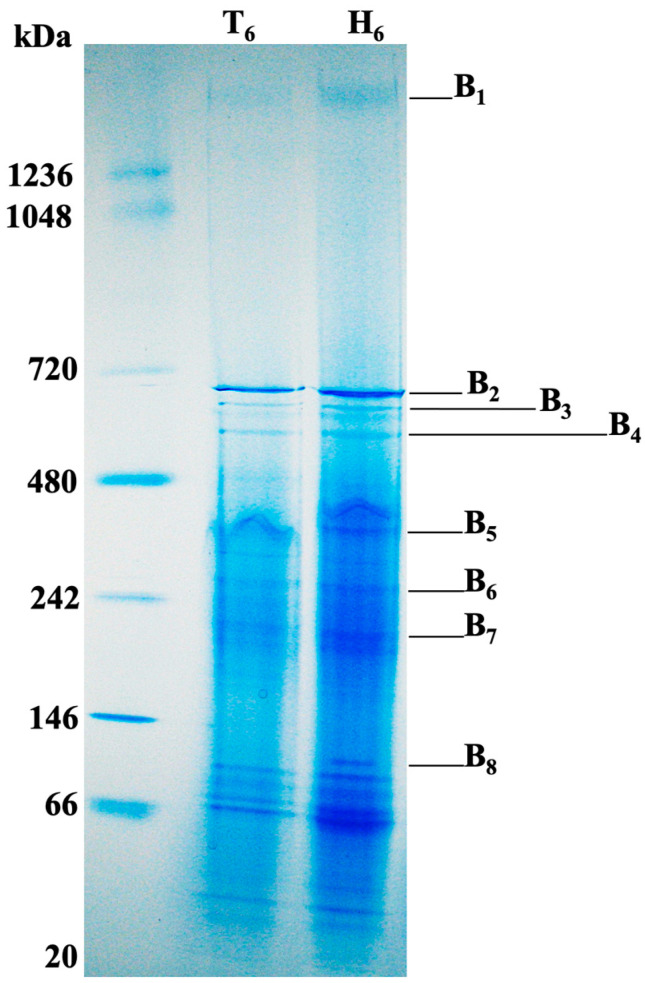
Membrane associated protein analysis of the bacterial lysogen IDH84/S1249 using BN-PAGE and LC/MS analyses. Approximately the same number of bacterial cells were collected after 6 h growth either in TSBYE broth (T_6_) or TSBYE with 50% human serum (H_6_), washed with PBS pH 7.4 and treated with 1% DDM to solubilize membrane-associated proteins in their native form, which were collected and separated using BN-PAGE. Eight different protein bands (B_1_-B_8_) showing differences in the lysogen when being exposed to human serum were excised for LC/MS analysis and identified proteins are described in Table 1.

Our early investigation indicated that the phage DNA detection in the supernatant significantly increased 6 h after human serum exposure [35], which is an indication of phage virion release. We, therefore, investigated membrane associated proteins of the lysogenic strain, particularly after 6 h exposure to human serum. Cells of the lysogen IDH84/S1249 were collected and treated with 1% DDM on ice to solubilize membrane protein complexes in their native forms, which were further separated using BN-PAGE analysis (Figure 4). Separated proteins, particularly eight protein bands with molecular masses of approximately 1.5 megadalton (mDa, B_1_), 700 kilodalton (kDa, B_2_), 680 kDa (B_3_), 600 kDa (B_4_), 400 kDa (B_5_), 300 kDa (B_6_), 200 kDa (B_7_), and 100 kDa (B_8_) showing differences in the lysogen when being exposed to human serum, were excised for LC/MS analyses. The top five detected bacterial proteins in highest abundance based on total spectrum counts (PSMs) averaged from two independent experiments are described in Table 1.

Among those most abundant identified bacterial proteins based on PSMs (Table 1), over 50% of them are related to carbohydrate metabolism and ATP production, which are highlighted in bold (Table 1). In addition, phage proteins, including hydrolytic enzyme endolysin, which targets bacterial peptidoglycan and lytic protein Rz, were also identified from the membrane of this lysogen after 6 h exposure to human serum (Table 1).

### 3.5. Bacterial Membrane Associated Protein Comparison between the Phage-Naïve Versus Its Isogenic Lysogenic Strain after Exposure to Human Serum

**Figure 5:**
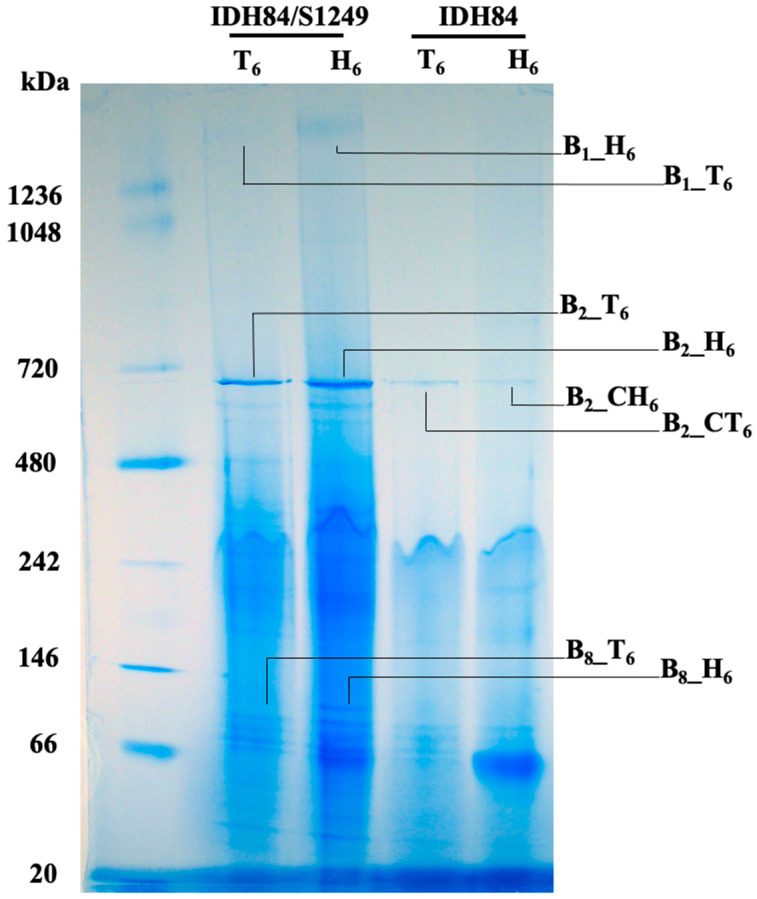
Bacterial membrane associated protein comparison between the phage-naïve strain IDH84 *versus* its isogenic lysogenic strain IDH84/S1249. Bacterial cells were collected after 6 h growth in either TSBYE broth (T_6_) or TSBYE with 50% human serum (H_6_), washed with PBS pH 7.4 and treated with 1% DDM. The solubilize membrane-associated proteins were collected from the supernatant and separated using BN-PAGE. The bacterial lysogen demonstrated significantly more DDM-solubilized proteins than the phage-naïve strain. Eight different protein bands (B_1__T_6_, B_1__H_6_; B_2__T_6_, B_2__H_6_, B_2__CT_6_, B_2__CH_6_; B_8__T_6_, B_8__H_6_), located at three molecular weights and associated with two different strains, were excised for semi-quantitative LC/MS analysis and data are described in Table 2.

Further analyses of the membrane proteins of the lysogen (IDH84/S1249) and its isogenic, phage-naïve strain (IDH84) demonstrated that significantly more proteins were released from the lysogenic strain (Figure 5). Eight different protein complexes (B_1__T_6_, B_1__H_6_; B_2__T_6_, B_2__H_6_, B_2__CT_6_, B_2__CH_6_; B_8__T_6_, B_8__H_6_), migrated at three positions (B_1_: 1.5 mDa, B_2_: 700 kDa, and B_8_: 100 kDa), associated with two different strains (lysogen: _T_6_ or _H_6_, phage-naïve strain: _CT_6_ or _CH_6_), and grown in two different conditions (T: TSBYE; H: TSBYE with 50% human serum) were excised for semi-quantitative LC/MS analyses described in Table 2.

Among those identified proteins, the multi-enzyme complex PDHc, composed of E1, E2, and E3, was detected with approximately 10-fold increase from the DDM-solubilized membrane sample of the lysogen, when grown with human serum (Figure 5 and Table 2: B_1__H_6_) compared to without serum (Figure 5 and Table 2: B_1__T_6_). The E1, E2, and E3 enzymes function consequently for oxidative decarboxylation of pyruvate, which generates acetyl-CoA, CO_2_, and NADH (H^+^), a gateway position in glucose metabolism to generate energy through aerobic expiration. In contrast, there was no obvious protein bands detected from the phage-naïve strain IDH84 at the same position of the BN-PAGE gel (Figure 5: IDH84) and LC/MS analyses of the gel slices excised from corresponding positions indicated no detected E1, E2, or E3, which indicated that the up-regulation of PDHc was regulated by the phage, directly or indirectly, when being exposed to human serum. In addition, the protein GroEL (Hsp60), an ATP-dependent molecular chaperonin essential for proper protein folding and biosynthesis, was co-isolated with PDHc and up-regulated over 10-fold in the serum-exposed lysogenic bacterial cells (Figure 5 and Table 2: B_1__H_6_) compared to the same lysogen grown without serum (Figure 5 and Table 2: B_1__T_6_).

The GroEL protein did not show any difference in the phage-naïve strain either with (Figure 5 and Table 2: B_2__CH_6_) or without human serum (Figure 5 and Table 2: B_2__CT_6_). In stark contrast, the GroEL detected from the lysogen demonstrated approximately 5-fold in the TSBYE (Figure 5 and Table 2: B_2__T_6_) and more than 7-fold increase in the presence of human serum (Figure 5 and Table 2: B_2__H_6_), compared to the phage-naïve strain (Figure 5 and Table 2: B_2__CT_6,_ B_2__CH_6_). The co-isolated proteins with GroEL were also different between the phage-naïve strain and its isogenic lysogenic strain (Table 2).

In addition to PDHc and GroEL, the elongation factors EF-Ts and EF-Tu, which are functioning in the peptide elongation during protein biosynthesis, were also found two-fold increase in the lysogen, when the lysogenic bacterial cells were grown in human serum (Figure 5 and Table 2: B_8__H_6_) compared to grown in TSBYE without serum (Figure 5 and Table 2: B_8__T_6_).

### 3.6. Membrane Protein Change Kinetics of the Lysogenic Bacterium Grown in Human Serum

**Figure 6:**
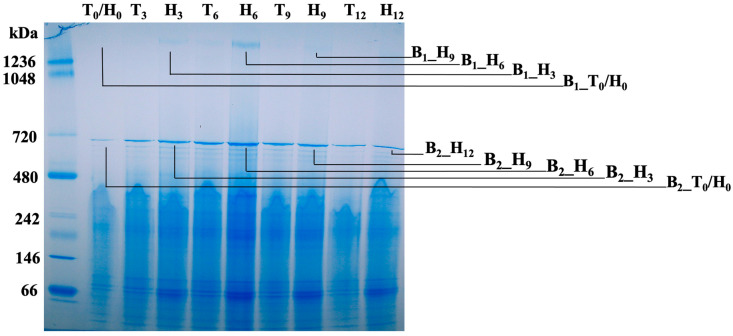
BN-PAGE analyses of membrane protein change kinetics of the lysogenic bacterium grown in human serum. T: TSBYE broth; H: TSBYE broth with 50% human serum; Numbers (0, 3, 6, 9, and 12) represent hours of incubation. Protein bands at two positions, B_1_ at approximately the 1.5 mDa position and B_2_ at the position of ~700 kDa, were detected from the lysogen with particularly strong signals after 6 h exposure in human serum. Four excised gel samples at the B_1_ position prepared as B_1__T_0_/H_o_, B_1__H_3_, B_1__H_6_, and B_1__H_9_, and five samples at the B_2_ position prepared as B_2__T_0_/H_o_, B_2__H_3_, B_2__H_6_, B_2__H_9_, and B2_H_12_, were collected for semi-quantitative LC/MS analysis and data are described in Table 3 and Table 4.

The BN-PAGE analysis of DDM-solubilized bacterial membrane protein complexes after different exposure time to human serum demonstrated that two protein complexes, which migrated at positions of 1.5 mDa and 700 kDa, were detected with changing densities over time, and showing the strongest signals after 6 h exposure (Figure 6). The B_1_ protein band at the position of 1.5 mDa and B_2_ band at the position of ~700 kDa, which are described in Figure 4 and Figure 5, were detected from the lysogen with particularly strong signals after 6 h exposure to human serum. Four excised gel slices at the B_1_ position, representing 0, 3, 6, and 9 h exposures to human serum labelled as B_1__T_0_/H_0_, B_1__H_3_, B_1__H_6_, and B_1__H_9_, and five gel slices at the B_2_ position representing 0, 3, 6, 9, and 12 h exposures to human serum labelled as B_2__T_0_/H_0_, B_2__H_3_, B_2__H_6_, B_2__H_9_, and B_2__H_12_, were collected for semi-quantitative LC/MS analysis (B_1_ protein bands: Table 3, and B_2_ protein bands: Table 4).

E1, E3, and E2 were detected with the most abundance after 6 h exposure to human serum (Table 3). Concomitantly, bacterial surface attached human serum proteins, including apolipoproteins B-100 (ApoB-100), ApoA-I, immunoglobulin heavy constant gamma (IGHG1), and immunoglobulin heavy constant mu (IGHM), were detected at the same time (Table 3). In addition to the peaked detection of E1, E2, and E3, the chaperonin GroEL, a key player in protein folding, was also detected with the most abundance after 6 h exposure to human serum (Table 4). Furthermore, human serum proteins, including IGHG1, IGHM, and keratin proteins, were increasingly detected in DDM-solubilized bacterial membrane proteins over time, which were likely bound to the bacterial membrane (Table 4).

## 4. Discussion

The 44 kb *Aggregatibacter* phage S1249 was originally identified from a clinical strain D11S-1, which can be isolated using mitomycin C induction [32,35]. Consistently, the same phage is also inducible in the newly generated lysogen DH84/S1249, as shown in Figure 1 and Figure 2. The lysogen demonstrated obvious recovery lagging after exposure to mitomycin C, compared to its isogenic, phage-naïve strain (Figure 1). This phage demonstrates a spheroid head with a diameter of approximate 60 nm and a tail structure of about 100 nm in length, including an inner rigid tube, an outer tail sheath and delicate tail fibers [35]. Those contractile tail features, which are also demonstrated here (Figure 2), attributes phage S1249 to the family of *Myoviridae*.

Our earlier work demonstrated that the total DNA synthesized by the phage S1249 infected bacterial lysogen peaked at approximately 6 h after exposure to human serum and, double amount of DNA was produced by the lysogen grown in human serum *versus* the TSBYE medium without serum [32,35]. However, a dramatical increase of cellular DNA was not due to the replication of the bacterial chromosomic DNA [32,35]. Concomitantly, phage DNA detected from the human serum culture spent medium showed five-fold increase after 6 h incubation in human serum [35], suggesting that virion progeny releases. Consistently, in the current study, the phage plaque assay demonstrated that phage S1249 formed 3- to 4-fold larger, clear plaques when being exposed to human serum, compared to turbid plaques formed on agars without serum or with sera from other animals, e.g., equine (Figure 3). Clear and larger plaques formed on the human serum agar indicate that phage S1249 is in the lytic state with increased burst sizes when being exposure to human serum. Together, the data imply that the accelerated DNA production by the lysogen within the first 6 h incubation in human serum is mainly due to phage replication. Phage burst sizes are found regulated by factors including temperatures [49] and antibiotics [50]. Nonetheless, further investigations are necessary to identify human serum components that control the lytic cycle determination and the burst size of the phage.

For better understanding phage virion release in vivo, the bacterial lysogen grown in serum culture media were collected at different time points for the analysis of bacterial membrane changes. The mild, non-ionized, detergent DDM was used to solubilize membrane protein complexes without disrupting protein–protein interactions, and protein complexes were further separated using blue native gel electrophoresis and analyzed using mass spectrometry. DDM breaks down the lipid–lipid or lipid–protein interaction, but maintains the solubilized proteins in their native forms, as well as preserving protein activities [45,46,51].

**Figure 7:**
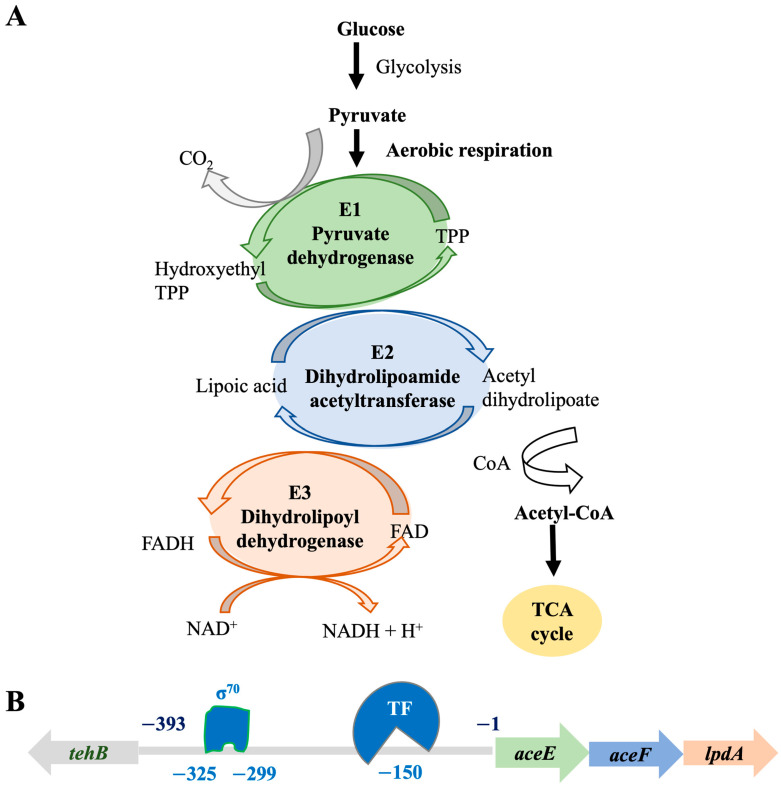
Hypothetical phage-mediated regulation of bacterial PDHc in the presence of human serum. The PDHc is formed by multiple copies of three enzymes: E1, E2, and E3, which catalyze the reaction of oxidative decarboxylation of pyruvate to acetyl-CoA, and connect glycolysis to TCA cycle, an important step to produce ATP through aerobic respiration. (**A**) PDHc catalytic reaction. Pyruvate + CoA + NAD^+^ → acetyl-CoA + CO_2_ + NADH. Thiamine pyrophosphate (TPP). (**B**) The PDHc operon in *A. actinomycetemcomitans*. The E1, E2, and E3 enzymes are encoded by a three-gene operon *aceE-aceF-lpdA* with a 393-bp intergenic region (IGR), up-streamed with the gene of *tehB* (Tellurite resistance protein B). In silico analysis of the IGR indicated a house keeping σ^70^ (−10 and −35 sequence locations) and, an alternative transcription factor (TF) binding site, which suggests transcriptional regulation of the PDHc operon.

The protein profiles of the lysogenic strain solubilized by 1% DDM were in stark difference, compared to its isogenic, phage-naïve strain (Figure 5). Serial samples were also collected from the phage-naïve strain, treated with DDM, and performed the BN-PAGE, which, however, showed significantly fewer solubilized proteins. The protein complexes separated by the blue native gel electrophoresis demonstrated that a protein complex band at the position of approximately1.5 mDa was different in the lysogen, particularly in the growth condition of human serum. Mass spectrometric analyses indicated that this 1.5 mDa protein band was mainly composed of three major enzymes: E1, E2, and E3, up-regulated approximately 10-fold in the presence of human serum (Figure 5, Table 2). These three enzymes work consequentially under aerobic respiration to convert pyruvate into acetyl-CoA, which will be used for TCA cycle to generate ATP (Figure 7A). On the contrary, E1, E2, and E3 of PDHc were not found up-regulated in the phage-naive strain in the presence of human serum (Figure 5: IDH84_H_6_). Up-regulation of PDHc indicates activation of aerobic respiration, leading to 18 times more ATP production compared to anaerobic respiration [39], which suggests the importance of efficient energy production for phage remaining in the lytic state.

An earlier work based on a lysogenic phage HK022 of *Escherichia coli* conducted by Carey et al. [52] demonstrated that phage infection and integration reprogramed bacterial respiratory system and inhibited bacterial aerobic respiration at the transcriptional level. The PDHc of *A. actinomycetemcomitans* (Figure 7B), formed by multiple copies of E1, E2, and E3, is encoded by a three-gene operon *aceE*-*aceF*-*lpdA* with an upstream 393-bp intergenic region (IGR). In silico analysis of the IGR sequence demonstrated cognate −10 and −35 box sequences for the regular house keeping σ^70^ binding, which are located at approximately the positions of −325 and −299, as well as potential alternative transcription factor binding site located close to the position of −150 (Figure 7B). Consistently, our early study demonstrated that bacterial alternative transcriptional factors (TF), including *rpoE-rseABC*, *rpoH,* and the global SOS system *recA-recX*, were up-regulated 2- to 1.5-fold [32].

In addition to bacterial transcription factors (BTF), human serum exposure of the lysogen also stimulated up regulation of phage regulatory factors [35], including two newly identified proteins: 52-amino-acid (aa) D11S_2259 (ID: 8672828) up-regulated 10-fold and 67-aa D11S_2260 (ID: 8672829) up-regulated 5-fold, as well as 146-aa D11S_2261 (ID: 8672830, a homologue of antitoxin SocA) up-regulated 2-fold, and 217-aa phage anti-repressor D11S_2263 (ID:8672832) up-regulated 2-fold in human serum [35]. Those identified phage regulatory proteins contain helical structures, based on AlphaFold prediction, and have the potential to bind to DNA and function as transcript factors, which may regulate PDHc directly by binding to the promoter sequence of PDHc. Or those phage regulatory proteins regulate bacterial PDHc indirectly, by regulating BTFs first, and the PDHc operon is presumably one of many regulons of the identified serum mediated BTFs, as describe before [32]. Together, it is likely that human serum components trigger the activation of phage transcription factors first, which reprograms bacterial metabolism and energy production, including PDHc, by hijacking bacterial alternative transcription factors (BTFs). The phage manipulated BTF binds to the promoter of PDHc to regulate pyruvate metabolism (Figure 7).

Furthermore, chaperone proteins, including GroEL (Hsp60), Dnak (Hsp70), and heat shock protein 90 (Hsp90), were found up-regulated in the lysogen (Table 1 and Table 2). These molecular chaperones are highly conserved across species of living cells, from bacteria to humans, and they act in an ATP-dependent manner and facilitate de novo protein folding, remodeling, oligomerization, and degradation to assure proper protein biosynthesis [53,54]. Chaperonin GroEL can be found in both bacteria and mitochondria of eukaryotic cells. GroEL and DnaK are among the most abundant chaperonins, which are essential for protein biosynthesis and, therefore, bacterial survivals and are also required for phage replication in bacteria [55,56]. They bind tremendously to their cofactors, distributing from bacterial cytosol, inner-membrane, periplasmic, to outer-membranes; and phage proteins as well [57]. Unlike GroEL and DnaK, Hsp90 is not essential in some bacteria [53], which demonstrates more selective interaction with its substrates [58,59]. Interestingly, the Hsp90 of mammalian cells has been identified facilitating viral infections [60,61]; nevertheless, the role of bacterial Hsp90 in phage infection and replication is unclear.

As an essential element for phage virion replication, GroEL was detected from the lysogen approximately 5-fold higher, compared to its isogenic, phage-naïve strain, and exposure to human serum, but not equine serum, further boosted the membrane-associated protein level of GroEL, but not the phage-naïve strain. The highly ATP-dependent chaperonin GroEL showed co-isolated with PDHc (Table 1, Table 2, Table 3 and Table 4), which is another indication of the importance of PDHc in the energy production and phage propagation in the serum-rich environment. Furthermore, the elongation factors (EF-Tu and EF-Ts) from the lysogen were also up regulated in the lysogen during exposure to human serum (Table 2). The highly conserved EF-Tu and EF-Ts of bacteria facilitate protein synthesis in the translation step using chaperone GTP as energy source to accelerate binding of aminoacyl-tRNA to ribosome [62], which is also ATP-dependent. All evidence here indicates that the phage reprograms its bacterial host metabolism and energy production, including the control of PDHc pathway, and switch into a lytic state during exposure to human serum.

In terms of what specific human serum components that might contribute to the phage life cycle switch and virions egress, we observed significantly increased numbers of apolipoproteins, which included the high-density lipoprotein ApoA-I and the low-density lipoprotein ApoB-100, and immunoglobulins including IGHG1 and IGHM detected from the membrane of the lysogen, particularly during the early stage when phages started to egress (Table 3). Nevertheless, steady increasing numbers of complement C3 and immunoglobulin IGHG1 were also found associated with the membrane of the lysogen. We, however, did not observe the same phenomenon of phage life cycle switch when equine serum was used. Therefore, these unique human serum components likely play roles in the modulation of phage life cycle decisions.

## 5. Conclusions

Together, the data here suggest that human serum induces phage S1249 to enter lytic cycle, which leads to bacterial lysis. The up-regulation of PDHc leads to a more efficient ATP production and may be required for the phage remaining in the lytic state in a serum-rich in vivo environment. Nonetheless, additional investigation is necessary to understand regulatory pathways of phage mediated bacterial PDHc activation in this capnophilic, facultative anaerobe in vivo, as well as human serum components that potentially participate in this regulation.

## Figures and Tables

**Table 1:** LC/MS analysis of DDM-solubilized membrane associated proteins in the lysogenic strain after six-hour growth in human serum.

Protein Bands *	Top Five Most Abundant Proteins Based on Total Spectrum Counts (PSMs **)	
	*A. actinomycetemcomitans*	Bacteriophage S1249 ***
B_1_	Molecular chaperone GroEL (Hsp60, 463) **E1 (252)** **** E3 (165) **E2 (149)** Pilus assembly protein TadG (42)	D11S_2223 (1)
B_2_	GroEL (Hsp60, 1651) DNA-directed RNA polymerase subunit β (RpoB, D11S_0063, 125) DNA-directed RNA polymerase (DdRP) subunit β (D11S_0064, 107) α-2-marcoglobulin (95) **Acetaldehyde dehydrogenase (85)**	D11S_2237 (4) D11S_2262 (1) D11S_2208 (1) D11S_2261 (1)
B_3_	**Malic enzyme (291)** **Citrate lysate (249)** DdRP subunit β (D11S_0064, 159) RpoB (D11S_0063, 145) **ATP synthase F0-F1 β (91)**	D11S_2237 (6)
B_4_	**ATP synthase F0-F1 β (260)** **ATP synthase F0-F1 α (195)** DdRP subunit β (D11S_0064, 183) RpoB (D11S_0063, 168) **Malic enzyme (94)**	D11S_2237 (8) D11S_2240 Endolysin protein (2) D11S_2239 Lytic protein R_z_ (2)
B_5_	**Fumarate hydratase (331)** **Pyruvate formate-lyase (PFL, 179)** **Na(+)-NQR subunit A (153)** Alanyl-tRNA synthetase (133) **Pyruvate kinase (120)**	D11S_2267 (3) Endolysin protein (2) Lytic protein R_z_ (2) D11S_2242 (2)
B_6_	**PFL (240)** Aspartate ammonia-lyase (170) **Glyceraldehyde 3-phosphate dehydrogenase (GAPDH, 129)** DnaK (Hsp70, 115) Aspartyl-tRNA synthetase (102)	D11S_2223 (5) D11S_2267 (3)
B_7_	**PFL (553)** **Fumarate reductase (Frd, 262)** **GAPDH (205)** Purine nucleoside phosphorylase (205) Preprotein translocase, SecA (178)	D11S_2267 (3) D11S_2260 (3)
B_8_	Elongation factor Ts (EF-Ts, 235) **Phosphoenolpyruvate carboxykinase (199)** Elongation factor Tu (EF-Tu, 187) DnaK (Hsp70, 148) Heat shock protein 90 (Hsp90, 122)	D11S_2258 (12) D11S_2267 (12) D11S_2257 (7) D11S_2228 (2)

* Identified protein bands were excised from BN-PAGE gels described in Figure 4. ** Total spectrum counts are based on average vales of peptide spectrum matches (**PSMs**) from two experiments. *** Identified phage protein locus tags were based on https://www.ncbi.nlm.nih.gov/nuccore/GQ866233.1. **** Enzymes that participate in sugar metabolism and ATP production are highlighted in bold.

**Table 2:** Comparative LC/MS analysis of membrane associated proteins of the lysogenic vs. phage-naïve strain, after grown with and without human serum for six hours.

Protein Bands *	Top Five Identified Proteins of *A. actinomycetemcomitans* (PSMs **)
B_1__T_6_	GroEL (34) *** E1 (18) E3 (18) E2 (15) Porin OmpA (4)
B_1__H_6_	GroEL (463) E1 (252) E3 (165) E2 (149) TadG (42)
B_2__CT_6_	GroEL (246) TadG (72) α-2-marcoglobulin (58) EF-Tu (53) EF-Ts (50)
B_2__CH_6_	GroEL (230) TadG (70) Transaldolase (31) Malic enzyme (31) Sugar ABC transporter substrate binding protein (31)
B_2__T_6_	GroEL (1193) α-2-marcoglobulin (145) RpoB (D11S_0063, 122) DdRP subunit β (D11S_0064, 90) Malic enzyme (77)
B_2__H_6_	GroEL (1651) RpoB (D11S_0063, 125) DdRP subunit β (D11S_0064, 107) α-2-marcoglobulin (95) Acetaldehyde dehydrogenase (85)
B_8__T_6_	Phosphoenolpyruvate carboxykinase (PEPCK, 167) EF-Ts (131) Heat shock protein 90 (127) EF-Tu (116) Meth_synth ^#^ (115)
B_8__H_6_	EF-Ts (275) EF-Tu (226) PEPCK (195) Heat shock protein 90 (131) DnaK (122)

* Identified protein bands were obtained from BN-PAGE gels described in Figure 5. ** Total spectrum counts highlighted in blue color are based on average values of **PSMs** from two individual experiments. *** Proteins highlighted in bold showed increased detections from the lysogen. ^#^ Meth_synth: 5-methyltetrahydropteroyltriglutamate--homocysteine methyltransferase.

**Table 3:** LC/MS analysis of membrane associated bacterial PDHc protein changes when exposed to human serum.

Protein Bands *	Top Five Identified Proteins (PSMs **)	
	*A. actinomycetemcomitans*	Attached Human Serum Proteins
B_1__T_0_/H_0_	GroEL (18) E1 (3) E3 (3) E2 (3) Hemolysin (3)	N/A
B_1__H_3_	E1 (94) GroEL (86) E3 (67) E2 (44) Hemolysin (14)	Keratin, type II cytoskeletal 1 (42) Keratin, type I cytoskeletal 9 (28) Keratin, type I cytoskeletal 10 (24) Keratin, type II cytoskeletal 2 epidermal (22) IGHG1 (22)
B_1__H_6_	GroEL (463) E1 (252) E3 (165) E2 (149) TadG (42)	Apolipoprotein B-100 (61) *** Apolipoprotein A-I (44) IGHG1 (38) IGHM (30) Keratin, type I cytoskeletal 9 (29)
B_1__H_9_	GroEL (102) E1 (71) E3 (42) E2 (39) Porin (23)	Apolipoprotein A-I (40) Keratin, type II cytoskeletal 2 epidermal (36) Apolipoprotein A-IV (32) Keratin, type II cytoskeletal 1 (30) Keratin, type I cytoskeletal 10 (29)

* Identified protein bands are based on the BN-PAGE gel described in Figure 6; N/A: Not applicable. ** Total spectrum counts in blue are based on average values of PSMs from duplicate experiments. *** E1, E3, and E2 were detected with the most abundance after 6 h incubation with attached apolipoproteins and immunoglobulins.

**Table 4:** LC/MS analysis of bacterial membrane protein change kinetics.

Protein Bands *	Top Five Identified Proteins (PSMs **)		
	*A. actinomycetemcomitans* ^1^	Attached Human Serum Proteins ^2^	Phage S1249 ***
B_2__T_0_/H_0_	Meth_synth (135) Elongation factor Tu (78) PEPCK ^#^ (68) Elongation factor Ts (65) Pilus assembly protein CpaF (43)	N/A	N/D
B_2__H_3_	GroEL (495) α-2-marcoglobulin (79) Citrate lyase subunit alpha (41) DdRP subunit β (D11S_0064, 39) Malic enzyme (39)	Keratin, type II cytoskeletal 1 (52) Haptoglobin (31) Keratin, type I cytoskeletal 10 (28) IGHG1 (25) Keratin, type I cytoskeletal 9 (24)	N/D
B_2__H_6_	GroEL (1651) **** RpoB (D11S_0063, 125) DdRP subunit β (D11S_0064, 107) α-2-marcoglobulin (95) Acetaldehyde dehydrogenase (85)	Complement C3 (29) IGHG1 (28) Serum albumin (21) Keratin, type II cytoskeletal 1 (21) Haptoglobin (20)	D11S_2237 (4) D11S_2262 (1) D11S_2208 (1) D11S_2261 (1)
B_2__H_9_	GroEL (502) α-2-marcoglobulin (97) TadG (49) Metallophosphatase (43) Malic enzyme (41)	Complement C3 (59) Keratin, type II cytoskeletal 1 (39) IGHG1 (37) Keratin, type I cytoskeletal 9 (29) Haptoglobin (26)	N/D
B_2__H_12_	GroEL (558) α-2-marcoglobulin (63) TadG (42) Acetaldehyde dehydrogenase (40) Malic enzyme (39)	Complement C3 (75) Keratin, type II cytoskeletal 1 (75) Keratin, type I cytoskeletal 9 (49) IGHG1 (49) Keratin, type II cytoskeletal 6C (42)	N/D

* Identified protein bands are based on the blue native gel electrophoresis, described in Figure 3. ** Total spectrum counts in blue are based on average values of **PSMs** from two individual experiments. *** Identified phage protein locus tags were based on https://www.ncbi.nlm.nih.gov/nuccore/GQ866233.1. **** GroEL demonstrated the most abundance in the lysogen after 6 h exposure with concomitant detection of phage proteins. N/D: not detected. ^#^ PEPCK: Phosphoenolpyruvate carboxykinase. ^1^ GroEL demonstrated the highest detection from the lysogen after 6 h exposure to human serum, with concomitant detection of phage proteins; ^2^ Complement C3 and IGHG1 attached to the lysogen over time.

## Data Availability

Important data are provided with the manuscript, and extra data are available upon request. The sequence databases have been deposited in public domains, including *A. actinomycetemcomitans* (https://www.ncbi.nlm.nih.gov/: CP001733) and phage S1249 (https://www.ncbi.nlm.nih.gov/: GQ866233), as well as Homo sapiens (https://www.uniprot.org/proteomes/UP000005640).
